# Supplementary material for: Comprehensive phase diagram of two-dimensional space charge doped Bi2Sr2CaCu2O8+x
Source: Nat Commun. 2017 Dec 12;8:2060. doi: 10.1038/s41467-017-02104-z (PMC5727194; doi:10.1038/s41467-017-02104-z)
Supplement: Supplementary file 1 — Supplementary Information [file 41467_2017_2104_MOESM1_ESM.pdf]

Supplementary Note 1: Space charge doping of the 1 u.c. BSCCO-2212 device

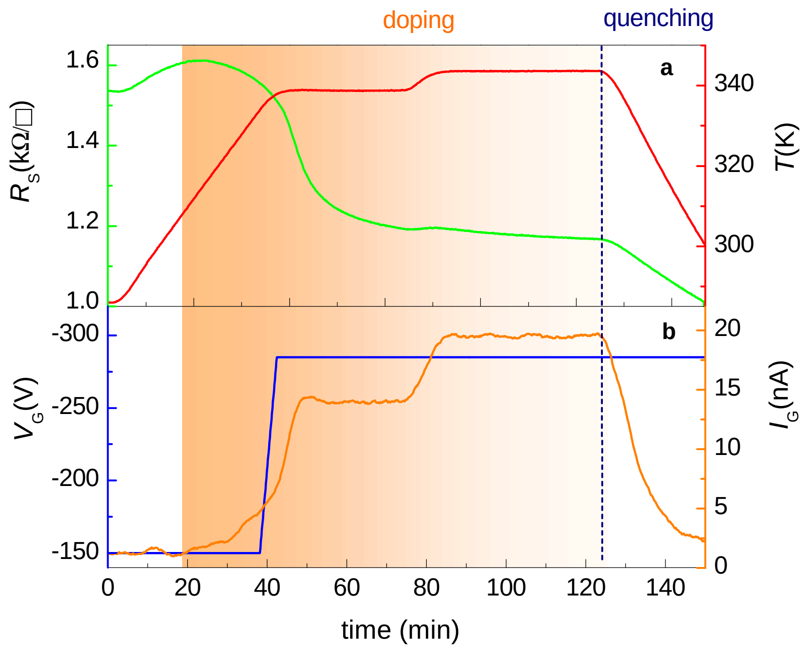

Supplementary figure 1: **Space charge doping dynamics. a**, Sheet resistance (green line, left axis) of the 1 u.c. BSCCO-device as a function of doping time. Temperature (red line, right axis) is increase up to 340-345 K and then decreased when the process is quenched. **b**, Applied gate voltage (blue line, left axis) and measured gate current (orange line, right axis).

In Supplementary figure **1**, the dynamics of the space charge doping technique is described by considering as example the 1 u.c. BSCCO-2212 device. The graph reports the behavior over time of the quantities involved in the doping process: V_{G} (gate voltage), T (temperature) R_{S} (sheet resistance) and I_{G} (gate current). V_{G} and T are the doping parameters, whereas R_{S} and I_{G} are the two quantities monitored during the doping process. At the beginning a negative voltage of -150 V is applied with the purpose of p-doping the sample. The doping process is highlighted with the orange area and begins once the temperature is sufficiently high to activate the Na^+ mobility within the glass ($T \simeq$ 315-320 K), as is possible to notice by the downward deviation of R_{S}(T) from its linear strange metal behavior. Once the temperature is stabilized at T_{d} (340-350 K), further and faster decrease in R_{S}(T) is achieved by increasing V_{G}. When the desired value of R_{S}(T) is reached the process is quenched by cooling the systems thus freezing the space charge in the proximity of the glass-material interface [1, 2].The high gate voltage used is due both to the high viscosity of

ions in hot glass and to the thickness of the glass dielectric substrate (0.5 mm). Estimating charge transfer by integrating the gate current would be useful but is not currently possible because of experimental constraints.

Supplementary Note 2: Hall measurements

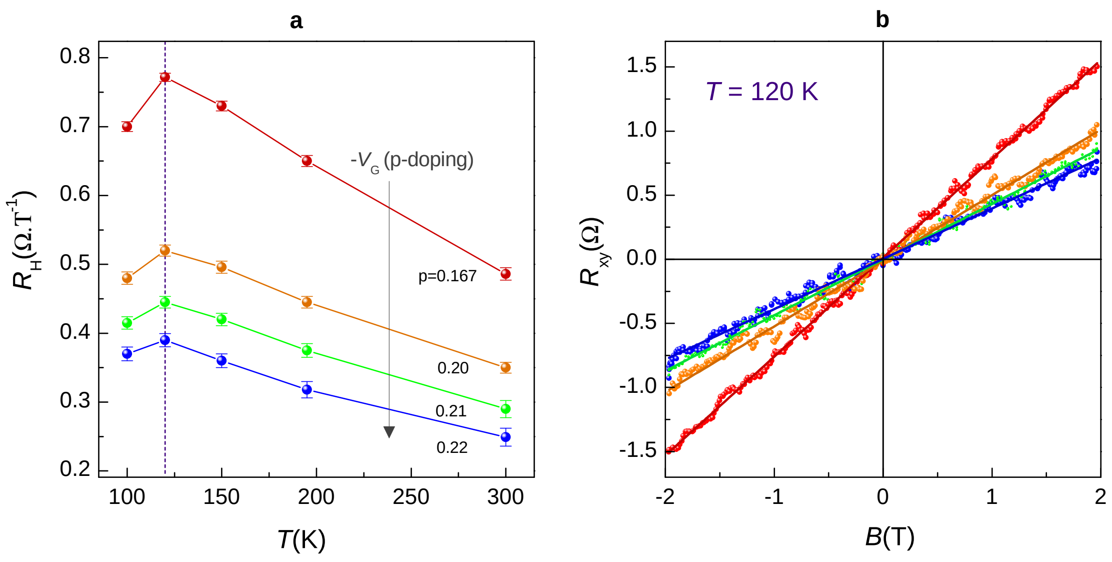

Supplementary figure 2: **Hall measurements. a**, Temperature dependence of the Hall coefficient R_{H} for different doping levels tuned by external gate voltage V_{G}. **b**, Transverse anti-symmetrized resistance R_{xy} as a function of magnetic field at $T = 120$ K. Colors correspond in the figures to the same doping level

We performed Hall measurements at different temperatures in order to confirm the anomalous dependence on temperature of the Hall coefficient R_{H} ([3, 4]) in our BSCCO-2212 thin films, as reported in the literature [5]. In supplementary figure **2**a the R_{H} vs T curves corresponding to different doping levels are shown. The maximum of R_{H}(T) is found at $T = 120$ K, and we consider these values of R_{H} to estimate the carrier density after each doping performed. Supplementary figure **2**b shows the anti-symmetrized transverse resistance R_{xy} as a function of applied magnetic field B at $T = 120$ K.

Supplementary Note 3: Reversibility of the doping process

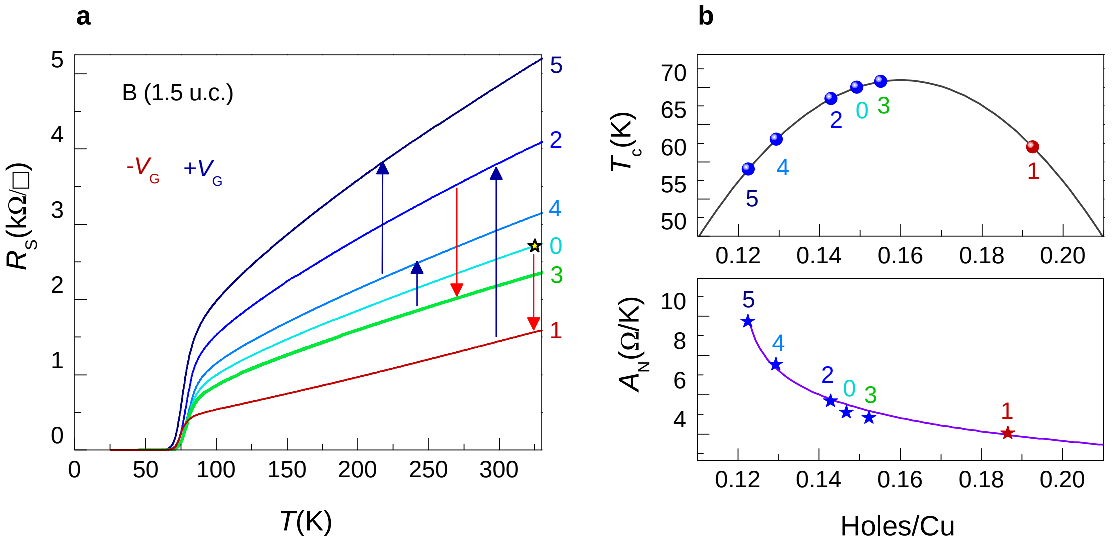

Supplementary figure 3: **Reversibility of the doping method a**, Sheet resistance vs temperature characteristics of sample B, where carrier density is modulated in a non monotonic fashion trough space charge doping method by applying positive (blue arrows) and negative voltages (red arrows) V_G . **b**, Tc (upper panel) and the slopes A_N (lower panel) of the linear part of R_S(T) at the several doping levels

In supplementary figure **3** we show an example of reversible modulation of the carrier density across the phase diagram. Supplementary figure **3**a shows a sequence of measurement from initial doping (0) to a final doping (5). Supplementary figure **3**b highlights the reversibility of the doping process by plotting T_c and the slope of the linear part of the resistivity curve as a function of doping. The doping was changed in a non-monotonic manner. The coherence and monotonic nature of these quantities again validates the reversibility of the process. Similar measurements of reversibility have been obtained on graphene ([2]) or MoS_2 ([1]) among others. We have not observed any electrochemical character to date on any sample or measurement.

Supplementary Note 4: Sample fabrication and storage

The samples are exposed to moderately hot temperatures during the fabrication process and eventually during the contact deposition process. We strive to reduce this temperature

as much as possible to avoid oxygen loss and sample degradation. After fabrication samples are stored in primary vacuum. Samples and devices A, B and D have been fabricated 2-4 days prior to insertion in high vacuum for measurement (10^{-6} mbar). Sample C, which had to be stored for a month in primary vacuum, suffered an initial T_c degradation, probably due to oxygen loss.

Supplementary Note 5: Determination of the characteristic temperatures

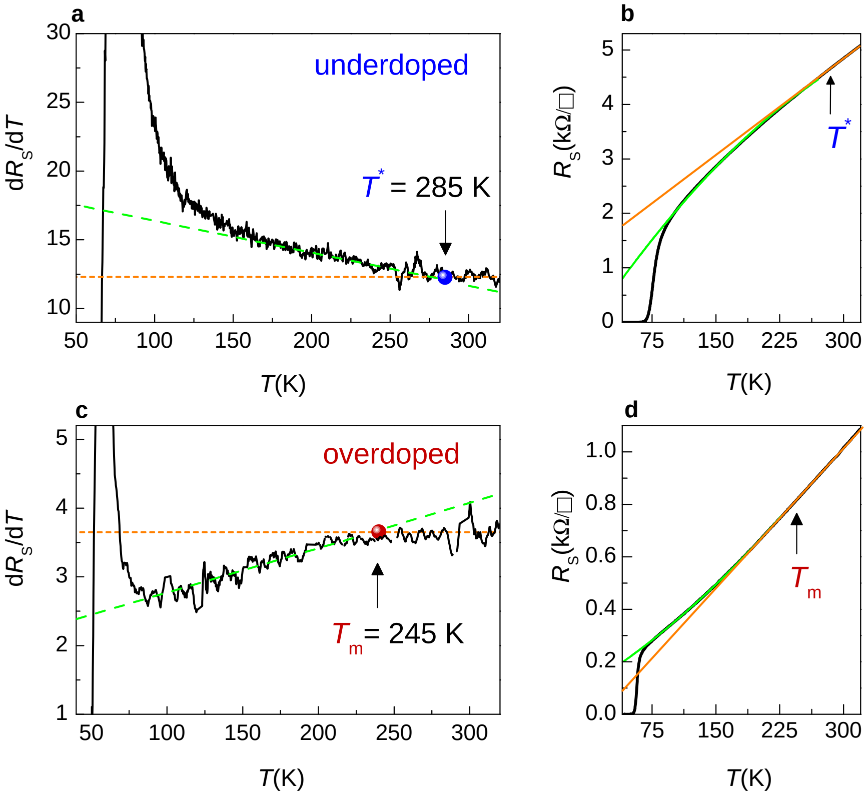

Supplementary figure 4: **Extraction of the characteristic temperatures. a, c** First order derivative of R_{S}(T) versus T for an underdoped and overdoped sample respectively. T^* (blue dot) and T_{m} (red dot) are found as the intersection between high temperature strange metal behavior (orange dashed lines) and the low temperature normal states (green dashed lines). **b, d**, Sheet resistance versus temperature curves corresponding to **a, c**. T^*, T_{m} determined as described in **a, c** coincide with the deflection and inflection points of low temperature normal states (green lines) with respect high temperature strange metal behavior (orange lines) respectively.

The extraction of T^* and T_m with the method explained in the main text was confirmed by analyzing the first order derivative curves dR_S/dT [6, 7]. In the dR_S/dT versus T plot, T^* and T_m are determined as the upward and downward deviation of dR_S/dT from its high temperature constant value (strange metal phase), whether the sample is underdoped or overdoped respectively as shown in supplementary figure **4**.

Supplementary Note 6: Screening length for electrostatic doping

Some estimations concerning screening length in cuprates for electrostatic doping do exist. Supplementary references [8, 9] indicate that in LSCO the screening length is sub nm, whereas references [10, 11] indicate that in YBCO it is of the order of 1-1.5 nm, and that it could be twice as much in BSCCO. Our data on BSCCO is in good agreement with this estimation because sample A which is 2.5 nm thick shows a small discrepancy in the Hall coefficient estimated doping value. If a gradient in doping exists, it would show up in the superconducting transition width and it is indeed seen only in sample A.

Supplementary References

[1] Biscaras, J., Chen, Z., Paradisi, A. & Shukla, A. Onset of two-dimensional superconductivity in space charge doped few-layer molybdenum disulfide. *Nature Communications* **6**, 8826 (2015).

[2] Paradisi, A., Biscaras, J. & Shukla, A. Space charge induced electrostatic doping of two-dimensional materials: Graphene as a case study. *Applied Physics Letters* **107**, 143103 (2015).

[3] Ando, Y., Kurita, Y., Komiya, S., Ono, S. & Segawa, K. Evolution of the Hall Coefficient and the Peculiar Electronic Structure of the Cuprate Superconductors. *Physical Review Letters* **92**, 197001 (2004).

[4] Bouvier, J. & Bok, J. Electron-Phonon Interaction in the High- Cuprates in the Framework of the Van Hove Scenario. *Advances in Condensed Matter Physics* **2010**, 472636 (2010).

[5] Bok, J. & Bouvier, J. Hall effect in the normal state of high-Tc cuprates. *Physica C: Superconductivity* **408-410**, 242–243 (2004).

[6] Barisic, N. *et al.* Universal sheet resistance and revised phase diagram of the cuprate high-temperature superconductors. *Proceedings of the National Academy of Sciences* **110**, 12235–12240 (2013).

[7] Ando, Y., Komiya, S., Segawa, K., Ono, S. & Kurita, Y. Electronic Phase Diagram of High-Tc Cuprate Superconductors from a Mapping of the In-Plane Resistivity Curvature. *Physical Review Letters* **93**, 267001 (2004).

[8] Smadici, S. *et al.* Superconducting Transition at 38 K in Insulating- Overdoped La_2CuO_4-$La_{1.64}Sr_0.36CuO_4$. *Physical Review Letters* **102**, 107004 (2009).

[9] Bollinger, A. T. *et al.* Superconductorinsulator transition in $La_{2-x}Sr_xCuO_4$ at the pair quantum resistance. *Nature* **472**, 458–460 (2011).

[10] Mannhart,J., Schlom, D.G., Muller, K.A. & Williams, E.J. Influence of the electric fields on pinning in $YBa_2Cu_3O_{7-\delta}$. *Physical Review Letters* **67**, 2099–2101 (1991).

[11] Frey,T., Mannhart,J., Bednorz, J.G. & Williams, E.J. Mechanism of the electric-field effect in the high-T_c cuprates. *Physical Review B* **51**, 3257–3260 (1994).
